# Societal costs of personality disorders among treatment-seeking patients in Norway: the relative contribution of specific DSM-5 categories

**DOI:** 10.1007/s00406-023-01655-1

**Published:** 2023-08-19

**Authors:** C. A. Sveen, G. Pedersen, D. A. Ulvestad, K. E. Zahl, T. Wilberg, E. H. Kvarstein

**Affiliations:** 1 Department of Child and Adolescent Psychiatry, Division of Mental Health and Addiction, Vestre Viken Hospital, Drammen, Norway; 2 Network for Personality Disorder, Section for Personality Psychiatry and Specialized Treatments, Department for National and Regional Functions, Division of Mental Health and Addiction, Oslo University Hospital, Oslo, Norway; 3 Norwegian Centre for Mental Disorders Research (NORMENT), Institute for Clinical Medicine, University of Oslo, Oslo, Norway; 4 Outpatient Clinic for Specialized Treatment of Personality Disorders, Section for Personality Psychiatry and Specialized Treatments, Department for National and Regional Functions, Division of Mental Health and Addiction, Oslo University Hospital, Oslo, Norway; 5 Group Therapy Section, Follo District Psychiatric Centre, Akershus University Hospital, Ski, Norway; 6 Section for Personality Psychiatry and Specialized Treatments, Department for National and Regional Functions, Division of Mental Health and Addiction, Oslo University Hospital, Oslo, Norway; 7 Institute of Clinical Medicine, University of Oslo, Oslo, Norway; 8 Section for Treatment Research, Department for Research and Innovation, Division of Mental Health and Addiction, Oslo University Hospital, Oslo, Norway

**Keywords:** Personality disorders, Societal costs, Regression analyses, Cost-of-illness

## Abstract

Personality disorders (PDs) are associated with high levels of societal costs, regardless of whether a single PD or a broad range of PDs have been studied. However, research on the relative contribution of specific PD-types on societal costs is limited. The aim of this study was to explore the possible contributions of the individual DSM-5 categories of PDs on the level of societal costs and its components (health service costs and productivity loss), while controlling for the impact of comorbid mental health and substance use disorders on these outcomes. Participants (*n* = 798) were retrieved from the quality register of the Norwegian Network for Personality Disorders—a collaboration of PD-treatment units within specialist mental health services. The patients were referred to treatment in the time-period 2017–2020. Costs were assessed using a structured interview covering the 6-month period prior to assessment. Diagnoses were determined by semi-structured diagnostic interviews (SCID-5-PD and M.I.N.I.). Statistics included multiple regression analyses. The main result was that no specific PD had a unique contribution to the high level of societal costs generally found among treatment-seeking patients with PDs. Borderline PD (BPD) was the only PD with significantly higher health service costs than the other PDs, while BPD, avoidant PD, and unspecified PD were independently associated with enhanced productivity loss. The differential cost-effects of specific PDs on the cost components were small. Several comorbid mental health and substance use disorders were significant contributors to costs, irrespective of PD status. The results underscore the importance of developing and implementing effective treatments for a broader range of PDs, to reduce the high levels of societal costs associated with all PDs.

## Introduction

In a recent cost-of-illness (COI) study in Norway, a substantial level of societal costs was demonstrated among treatment-seeking patients with a broad range of DSM-5 personality disorders (PDs), comparable to the societal costs of schizophrenia, and significantly higher than the societal costs of both depression and anxiety disorders [1]. The societal cost estimates converged with recent, register-based COI studies of borderline personality disorder (BPD) and schizotypal PD, but exceeded previous findings from other bottom-up studies, primarily focusing on BPD [2–8].

PDs are generally characterized by enduring maladaptive patterns of behavior, cognition, and inner experience, exhibited across many contexts and deviating from those accepted by the individual's culture [9]. In addition, patients with a variety of specific PD-types have different, partly contrasting presentations, ranging, for example, from personality problems of disinhibition, impulsivity, and dependency of others to the more introvert aspects with emotional restrictiveness, social inhibition, and avoidance [10]. A heterogeneity of presentations is commonly encountered within health services. Variation includes different types of PDs, comorbidity of PDs and comorbidity of other co-occurring mental health and substance use disorders (previously called Axis I disorders in DSM-IV) [11].

How individual differences of specific PDs are reflected in the level of societal costs and its components (health service costs and productivity loss), is not well investigated. To our knowledge, only two COI studies have used regression analyses to study the specific contributions of a broad range of PDs [2, 12]. One study investigated the individual contribution of a broad range of PDs on societal costs, direct medical costs and indirect costs among 1740 treatment-seeking patients [2], while the other study investigated the individual contribution of BPD, avoidant PD, unspecified PD, and depressive PD among 131 treatment-seeking adolescents on direct medical costs [12].

The aim of the present study was to explore the possible contributions of the individual DSM-5 categories of PDs among treatment-seeking patients on the level of societal costs and its components, while controlling for the impact of comorbid mental health and substance use disorders on these outcomes.

## Methods

### Setting and recruitment

The present study is based on data for the period 2017–2020 retrieved from the quality register of the Norwegian Network for Personality Disorders (Network), a nationwide clinical research collaboration [13, 14]. The present study included 15 different outpatient treatment units within the Network, which offer specialized treatment for adult patients with a variety of PDs or clinically relevant, subthreshold personality difficulties. Patients are referred to specialized PD-treatment from regular outpatient clinics, where an initial assessment of patients referred from general practitioners to specialist mental health service level is performed. Patients with comorbid psychosis, bipolar I disorder, autism, mental disability, and severe substance use disorders are not considered eligible for the PD-treatment programs, but a minor proportion may nonetheless be referred. The treatment units comprise multidisciplinary teams with different healthcare professionals including psychiatrists, psychologists, psychiatric nurses, social and occupational therapists. All units within the Network follow the same assessment procedures, using standard evaluation instruments and diagnostic interviews. Treatment approaches include specialized programs tailored to BPD (e.g., mentalization based therapy (MBT), dialectical behavior therapy (DBT), schema-focused therapy) as well as other treatments for PDs, such as psychodynamic group therapy, metacognitive interpersonal therapy, art therapy, body awareness therapy and groups focusing on psychoeducation [1].

### Participants

For the period 2017–2020, 798 patients in the Network’s quality register were assessed for both PDs and comorbid mental health and substance use disorders, and had completed the specific interview of health and welfare service use and occupational activity (“cost interview”), which was necessary to be included in the regression analyses.

In the study sample of 798 patients, 24.6% were male and 75.4% were female, and the mean age was 30.0 (SD = 8.9, range 18–63 years). A total of 639 patients (80.1%) had at least one PD diagnosis, of which 59.4% had only one PD diagnosis, 14.5% had two diagnoses, 4.8% had three diagnoses, 1.1% had four diagnoses, and 0.3% had five diagnoses. The remaining 159 patients (19.9%) had no PD diagnosis, but their mean number of fulfilled PD criteria was 3.5 (SD: 2.5). All patients were included in the regression analyses. Table 1 presents the distribution of PDs in the study sample.

**Table 1:** Distribution of personality disorders

	Frequency	Percent
No diagnosis	159	19.9
Unspecified	102	12.8
Schizoid	9	1.1
Schizotypal	3	0.4
Paranoid	68	8.5
Antisocial	19	2.4
Narcissistic	3	0.4
Borderline	267	33.5
Histrionic	5	0.6
Avoidant	291	36.5
Dependent	41	5.1
Obsessive–compulsive	58	7.3

*N* = 798. As patients can be diagnosed with more than one diagnosis of PD, the percentages will add up to more than 100%

Nearly all (94.7%) of the assessed patients were given at least one mental health and substance use disorder diagnosis (mean = 2.02, SD = 1.30). Most individual diagnoses were aggregated into categories, and the five most frequent categories were used as covariates in the regression analyses: Mood disorders, anxiety disorders, substance use disorders, eating disorders, and PTSD. The omitted diagnoses (somatoform disorder, dissociative disorder, ADHD, psychosis disorders, and autism spectrum disorder) had too few incidents (< 8%) to warrant inclusion as covariates in the analyses. Table 2 describes the number of patients in the different mental health and substance use categories.

**Table 2:** Categories of mental health and substance use disorders

	Frequency	Percent
No diagnosis	42	5.3
Mood disorders	560	70.2
Anxiety disorders	415	52.0
PTSD	107	13.4
Substance use disorders	83	10.4
Eating disorders	73	9.1

*N* = 798. As patients can be diagnosed with more than one diagnosis of mental health and substance use disorders, and thereby could be included in more than one category, the percentages will add up to more than 100%

### Diagnostic assessment

Systematic diagnostic evaluation was part of the initial assessment procedure on referral to all treatment units. In accordance with the DSM-5 [9], standardized, semi-structured diagnostic interviews were used; the Structured Clinical Interview for DSM-5 Personality Disorders for PD (SCID-5-PD) [15], and the Mini International Neuropsychiatric Interview (M.I.N.I.) [16, 17] for mental health and substance use disorders.

As first defined in the DSM-III [18], PD—not otherwise specified (PD-NOS) is indicated when the general criteria for PD is fulfilled, but criteria are below the threshold of any specific PD. The diagnosis is either set directly by the clinicians or set by the researchers according to a given set of criteria. The operationalization of PD-NOS lacks precision, and former studies have suggested cut-offs ranging from 5–11 fulfilled PD criteria across categories [19–23]. In line with a former study of PD-NOS and its operational definition in the Network, yielding comparable prevalence rates as in clinical samples reported in the meta-analyses of Verheul and Widiger, we chose to categorize patients with eight or more fulfilled PD criteria and no specific PDs as unspecified PD (based on SCID-5-PD and DSM-5 terminology), if not already given the diagnosis by the clinicians [1, 20, 24].

Diagnostic reliability was not directly investigated in this study. However, within the Network, diagnostic assessments were performed in each unit by clinical therapists who had received systematic training in diagnostic interviews and principles of the LEAD-procedure (Longitudinal, Expert, All-Data) [21, 25]. This means that diagnoses were based on all available information including referral letters, self-reported history and complaints, and overall clinical impression, in addition to the diagnostic interviews. All diagnoses were set or evaluated by a specialist in psychiatry or clinical psychology. In the study period, local training courses/workshops focusing on understanding and assessment of PDs, associated comorbidity, and use of structured interviews were conducted by an experienced psychiatrist (last author) at all units in order to ensure clinical competence and calibrate diagnostic evaluation. A total of 29 local workshops were held within the study period in addition to shorter clinical discussions on request [1]. Furthermore, in a former study using the Structured Clinical Interview for DSM-IV (SCID-II—the previous version of SCID-5-PD) within the Network, reliability was investigated and acceptable diagnostic reliability was indicated [26].

### Cost measures

Societal costs are the sum of direct and indirect costs. Direct costs cover all actual costs of healthcare utilization (general practitioner visits, emergency health services, outpatient treatment, medication, and both somatic and mental health inpatient treatment). Indirect costs cover the lost productivity due to suffering from PD. Intangible costs (i.e., the psychological pain experienced by people with PDs) are not included in societal costs in this study, as such costs are very difficult to measure [27]. Hence, the societal costs in this study are the sum of direct healthcare costs and productivity loss. Calculations of healthcare costs and productivity loss for the total period of six months prior to evaluation were estimated using a bottom-up approach [27], that is, taking the individual patients’ reported health service use and degree of absenteeism from the labor market, and multiplying it with the estimated unit cost of each specific cost-element [1].

Clinicians performed the cost interview as a part of the pretreatment assessment, collecting data for the 6-month period prior to assessment. Questions on health-service use included: (1) general practitioner (GP) visits; (2) emergency health services (psychiatric emergency helpline, emergency room, psychiatric outpatient emergency service, and ambulant emergency service); (3) hospitalization (admission to medical hospital, admission to psychiatric hospital, admission to addiction clinics, and day-patient care); (4) outpatient treatment at mental health centers (individual- or group therapy); and (5) pharmacological treatment. The participants were also asked to which degree they were employed the last 6 months (range 0–6).

All unit costs were measured in €, yearend 2018. Unit cost for GP was estimated based on a public report that estimated the total cost of all GPs [28] in 2017, adjusted by the official consumer price index (CPI) [29], divided by the total number of consultations by GPs during 2018 [30]. Unit cost for psychiatric emergency helpline was calculated based on the annual report 2018 from “Mental Helse”, a typical helpline provider in Norway [31]. The total cost of the service was divided by the total number of telephone calls (answered), chat-service and mail service. Emergency room unit cost was set to the price for same day consultation with specialist medical doctor at a private healthcare center in Oslo [32]. Psychiatric emergency outpatient service was set at the same unit cost as standard outpatient consultation, while emergency consultation at the patients home out of an outpatient clinic were given an ambulatory fee add on [33]. Unit costs related to treatment at outpatient mental health centers, medical and psychiatric hospitals, addiction clinics, and day-patient care were obtained from reports published by the Norwegian government [34, 35]. 

### Methods section

Calculations of medication unit costs were based on information from the Norwegian Medicines Agency, and cost per daily dose of typical drugs per medication class were used to calculate cost per month [36].

The human capital approach was used to calculate the productivity loss, as most COI studies have used this approach to estimate productivity loss [37, 38]. The human capital approach measures lost productivity as the patients’ absence from work due to illness, valued at the market wage. As the patients did not report their individual gross income, and the marked wage of patients with PDs are not available in public registers, the patients’ unit cost had to be estimated. As many patients with PDs struggle to stay in the workforce and achieve higher levels of education, the average monthly wage for the total population probably is an overestimation of the wage-level of patients with PDs (only 11.5% of the sample reports they have been in ordinary employment during the whole six months, while 72.6% of the sample reports no connection to the labor marked during the same period). The unit cost of lost productivity was thus set to be equal to the average monthly sickness benefit [39], which is 58% of the average monthly wage in Norway [40].

All unit costs, mean health service costs, mean productivity loss, and mean societal costs in the period 6 months prior to assessment are reported in detail by Sveen and colleagues in their COI study of treatment-seeking patients with PDs, using data from the quality register for the same period as the present study [1].

### Ethics

All participating patients from each treatment unit gave their written consent to use anonymous clinical data for research purposes. Anonymized data were collected and transferred to the quality register. The collection procedures were approved by a local data protection officer at each contributing unit. Data security procedures for the quality register were approved by the data protection officer at the research center of the Network at Oslo University Hospital. Because the data were anonymous, formal approval from the Norwegian State Data Inspectorate and Regional Committee for Medical Research and Ethics was not required.

### Statistical analysis

Multiple regression main effect analyses were performed in order to investigate the unique contribution of each type of PD, while controlling for the effects of the categories of comorbid mental health and substance use disorders, on (I) health service costs, (II) productivity loss, and (III) societal costs, respectively (in three separate analyses). Differences in age and gender may be associated with health service use [2, 41]. A preliminary regression analyses including age and gender in all three models showed non-significant effects (*p*-values in the range 0.25–0.85). In order to keep the models parsimonious, age and gender were omitted. The regression models thus included 16 independent variables. Due to the exploratory nature of the current investigation, no general adjustments for multiple comparisons were strictly required, and an alpha level of 0.05 was used to determine statistical significance for all analyses [42]. Table 3 presents exact *p*-values, and power analyses were conducted post hoc. The correlation matrix between all the independent variables as well as the Tolerance and Variance Inflation Factor (VIF) coefficients gave no indication of a multicollinearity problem in any of the models.

**Table 3:** Specific personality disorders as predictors of health service costs, productivity loss, and societal costs

Independent variable	Model I; health service costs		Model II; productivity loss		Model III; societal costs	
	β	*p*	β	*p*	β	*p*
Unspecified	0.022	0.596	0.102	0.014	0.022	0.592
Schizoid	− 0.016	0.664	0.006	0.871	− 0.016	0.669
Schizotypal	− 0.064	0.078	0.037	0.306	0.000	0.994
Paranoid	− 0.046	0.237	− 0.034	0.389	− 0.004	0.911
Antisocial	− 0.041	0.270	0.028	0.454	0.023	0.535
Narcissistic	− 0.027	0.471	0.067	0.078	0.008	0.831
Borderline	0.089	0.030	0.095	0.022	0.019	0.647
Histrionic	− 0.011	0.764	− 0.068	0.075	− 0.033	0.386
Avoidant	0.045	0.273	0.108	0.010	0.031	0.463
Dependent	0.042	0.255	− 0.033	0.380	− 0.029	0.436
Obsessive–compulsive	0.014	0.707	− 0.018	0.623	− 0.031	0.415
Mood disorders	0.095	0.010	− 0.062	0.094	0.000	0.999
Anxiety disorders	0.009	0.812	0.002	0.957	0.010	0.786
Substance use disorders	0.104	0.006	0.001	0.985	0.043	0.266
Eating disorders	0.093	0.011	− 0.092	0.013	0.136	< 0.001
PTSD	0.063	0.091	0.052	0.165	0.069	0.066

Covariates of mental health and substance use disorders are also included in the table. *N* = 744 (54 patients had missing data for at least one diagnostic category). Log-transformed dependent variable in model I, Beta weights and *p*-values for all models

All cost data in the present study were non-normally distributed. Most patients had similar health service costs, but a small proportion of patients had very high costs due to inpatient admissions. As many as 75% of the patients had been out of the workforce during all 6 months, incurring a large productivity loss, while only 10.3% had no productivity loss. Societal costs, the sum of health service costs and productivity loss, was accordingly non-normally distributed. In multiple regression, the assumption requiring a normal distribution, in order to make inferences about the population parameters, applies only to the residuals. They were all non-normally distributed as well. However, when the sample size is sufficiently large, like in the present sample, the Central Limit Theorem (CLT) ensures that the distributions of parameter estimates will approximate normality when the errors are independent and identically distributed with finite variance, regardless of the shape of the population distribution [43–45]. As the effect of CLT is moderated by the extent of non-normality in the population, a transformation which could improve the normality of the residuals significantly is nonetheless recommended [43]. Accordingly, we used a log-transformed health service cost model, as the residuals approximated normality after the transformation. The residuals of the other cost variables were not improved significantly by log transformations, hence the non-transformed models of both productivity loss and societal costs were used in the regression analyses. Statistical analyses were performed using SPSS version 28, except for the power analyses, which were performed using the R “pwr” package (version 1.3–0).

## Results

### Health service costs

Table 3 shows that BPD is the only PD with a unique contribution to total health service costs (*p* = 0.030). Further analyses showed that emergency use services was the only cost component significantly higher for BPD than the other PDs. Moreover, comorbid mood disorders (*p* = 0.010), substance use disorders (*p* = 0.006), and eating disorders (*p* = 0.011) had unique contributions of enhanced health service costs. The proportion of variance in total health service costs explained by model I was 5.2% (*R*^2^ = 0.052), and the overall model was significant (*p* = 0.001). Given the sample size, a significance level of 0.05, the number of independent variables, and the level of *R*^2^, the statistical power of this regression model was high (0.994). The beta weights (effect sizes) were small (close to 0.1 for all significant variables).

### Productivity loss

The specific PDs with unique cost-level contributions to productivity loss were BPD (*p* = 0.022), avoidant PD (*p* = 0.010), and unspecified PD (*p* = 0.014); see Table 3. Further analyses showed that the most frequent diagnostic criteria met for patients diagnosed with unspecified PD were from BPD and avoidant PD. Eating disorder was the only comorbid mental health disorder with a unique cost-level contribution (*p* = 0.013). BPD, avoidant PD, and unspecified PD had positive contributions to productivity loss, whereas eating disorders displayed a negative contribution. The proportion of explained variance in productivity loss by model II was 3.8% (*R*^2^ = 0.038), and the overall model was significant (*p* = 0.026). Given the sample size, a significance level of 0.05, the number of independent variables, and the level of *R*^2^, the statistical power of this regression model was high (0.953). The beta weights in this model were small (close to 0.1 for all significant variables).

### Societal costs

In this model no specific PD had a unique contribution to societal costs, and eating disorders was the only significant variable, uniquely contributing to an enhanced level of societal costs (*p* < 0.001); see Table 3. The proportion explained variance in societal costs by model III was 3.0% (*R*^2^ = 0.030), but the overall model was not significant (*p* = 0.140^1^). Given the sample size, a significance level of 0.05, the number of independent variables, and the level of *R*^2^, the statistical power of this regression model was 0.874. The beta weights in this model were generally very small, except for eating disorders with a still small, but somewhat higher beta weight than the other independent variables.

## Discussion

This study is a further elaboration of a large COI study in Norway, which demonstrated a substantial level of societal costs among a broad range of PDs [1]. Based on the same sample, the possible unique contributions of each specific PD on societal costs and its components were explored in the present study.

### Main findings

#### Health service costs

It is noteworthy that BPD was the only PD associated with enhanced levels of health service costs, mainly due to relatively high levels of emergency service use in the 6-month period before referral to treatment. Correspondingly, former trials of BPD treatments point to high levels of emergency services use, suicide attempts, and self-harming behaviors before starting treatment [46–49]. Few have investigated the relative contribution of BPD compared to other PDs, but similar results are reported in other studies of adult samples [2, 50], while another study found that no specific PD had a unique contribution on direct medical costs among adolescents (when including comorbid mental health and substance use disorders in the regression analysis). Overall, the finding of relatively high health service costs for BPD is in line with the majority of other BPD studies.

For patients with BPD, several studies have demonstrated how costly emergency and inpatient services can be reduced by well-tailored treatments, for example, DBT and MBT [49]. Our study, with all patients referred to treatment, do indeed confirm the relevance of such implementation within BPD healthcare organization.

Our study also demonstrates that the differential effect of BPD versus other PD diagnoses was rather small. For patients with other PDs, the generally high level of health service costs in the sample should therefore not be underestimated [1]. The treatment literature on effective treatments for other PDs is sparse, with only a few systematic reviews and limited evidence on psychological interventions [51, 52]. However, refinements of specialized approaches adjusted for other PDs are increasingly reported [53–56].

Among the major categories of mental health and substance use disorders included in the model as covariates, mood disorders, substance use disorders, and eating disorders were associated with increased total health service costs. Major depression and bipolar disorder are subcategories of mood disorders, and as comorbid conditions among PD patients it is likely that they will further enhance suicidal behavior, need for emergency services, and prolonged periods of inpatient treatment. Several studies have demonstrated greater severity of mood disorder in combination with PD [57–60]. Patients with comorbid substance use disorders or eating disorders may often need inpatient treatment as well, and such comorbidity renders an enhanced risk of somatic complications and impaired physical health, further reinforcing the need for inpatient treatment. The inpatient treatment for these comorbidities may also be for substantial periods of time, which yields higher cost levels. The present study thus demonstrates consequences of the complex picture of comorbidity, which is known to be characteristic in clinical PD samples [61–63]. The enhanced levels of health service costs associated with eating disorders, mood disorders, and substance use disorders, irrespective of PD status, highlights that the overall economic health service burden of patients with PDs also depends on the severity of condition in terms of the complicating presence of other mental health and substance use disorders.

The statistical inferences made from this model have a rather solid basis, due to the relatively low *p*-values among the significant variables, the high level of overall significance of the model, and the high level of statistical power. Even so, the contribution of the individual variables on health service costs, as indicated by the beta weights, was rather small.

#### Productivity loss

An important finding was that BPD, avoidant PD, and unspecified PD were the only PDs associated with enhanced productivity loss. Instability and dysfunction in affective, behavioral and interpersonal domains characterize BPD [64], and is likely to contribute to enhanced levels of productivity loss for this group. Several other studies have correspondingly found substantial social and occupational impairment among patients with BPD [65, 66]. Moreover, as BPD often starts in adolescence, it may affect educational levels and early establishment within the workforce [67, 68]. To our knowledge, only one former COI study has reported the results of a regression analysis including a broad range of PDs as independent variables, and BPD was associated with increased indirect costs in this study [2].

In our study, a novel finding was that avoidant PD was associated with elevated levels of productivity loss. The finding may not be surprising, as the patient group is characterized by excessive social anxiety, inhibition, and avoidance, and one of the specific criteria in DSM-5 is avoidance of occupational activities which involve significant interpersonal contact due to fear of criticism, disapproval, or rejection [9]. Several other studies have also indicated extensive psychosocial and occupational impairment, isolation, and poor life quality among patients with avoidant PD [69–72]. However, in the study by Soeteman and colleagues, avoidant PD was not associated with increased levels of productivity loss [2].

Perhaps more surprising was the finding that unspecified PD was the third and last variable associated with a unique contribution to productivity loss, as the few studies focusing on unspecified PD point to psychosocial impairment somewhat less severe or similar to patients with a specific PD [20, 22, 23]. However, it should be noted that criteria from BPD and avoidant PD were the most frequent diagnostic criteria met for the patients diagnosed with unspecified PD in our sample. This could give a possible explanation of the corresponding results found for BPD, avoidant PD and unspecified PD.

A noteworthy observation was also that eating disorders were associated with reduced productivity loss. The finding is surprising, and contrasts findings of Streatfeild and colleagues, who reported substantial levels of economic costs of eating disorders in the United States, with 75% of the costs being due to productivity loss [73]. In the present sample of treatment-seeking patients with PDs, comorbid eating disorders are generally of limited severity and functional impairment, and further subgroup analysis found that the reduced productivity loss was associated with the subcategories of Eating Disorder Not Otherwise Specified and Bulimia Nervosa, but not with Anorexia Nervosa. Even so, the reduced productivity loss associated with the less severe eating disorders is difficult to explain, and further research is needed.

Effect sizes were rather small in this model, whereas *p*-values of the overall model and the individual significant variables were well below the alpha level, and the power was high. On the other hand, the regression model of productivity loss was based on a rather skewed, kurtotic and bimodal dependent variable, with non-normal residuals. Hence, the effect of a large N and the CLT is uncertain in this case, and the results should be interpreted with some caution.

#### Societal costs

Societal costs have been found to be substantial among treatment-seeking patients with a broad range of DSM-5 PDs [1], comparable to the societal costs of schizophrenia, and significantly higher than the societal costs of both depression and anxiety disorders. Similar results were found for both BPD and schizotypal disorder in recent register based COI studies (each study investigating one specific PD) [4, 7]. In the present study, exploring the relative contributions of all DSM-5 PDs, including five covariate mental health and substance use categories, no specific PD had a unique contribution to societal costs. Power analyses revealed a relatively low probability of committing a type II error, and indicated that the lack of significant PD-types is a fairly robust result in this model. This is however contrary to the findings in the study by Soeteman and colleagues, where both BPD and obsessive–compulsive PD were uniquely associated with increased mean societal costs [74]. Although the present study and the study by Soeteman and colleagues have commonalities, there are some differences which may have had an impact on results. The number of participants was larger in their study (*N* = 1740), and it lacked covariates of mental health and substance use disorders. The level of *R*^2^ is comparable between the studies (2.4% in their study, 3.0% in the present study), indicating this is an expected level of explained variance in such comprehensive models. In sum, the finding that no specific PDs were associated with increased societal costs seems fairly robust. However, as the results are conflicting with the only other study using regression analysis to study the contribution of specific PDs, further research is necessary in this field.

Our model included comorbid mental health and substance use disorders in addition to PDs, and the results indicated a possible unique contribution of eating disorders. However, the fact that the overall regression model was not a significant predictor of societal costs (*p* = 0.140) is contrary to this finding, and it must be interpreted with a high level of caution.

### Strengths and limitations

A major strength of this study is the high number of participants, with data collected nationwide in different settings, hence enhancing the external validity of the results. Furthermore, the high number of participants allowed all types of PDs according to DSM-5 to be included, widening the scope of this investigation. However, it should be noted that the relatively infrequent PDs are statistically less likely to emerge as significant individual predictor diagnoses. Another strength of the study is the inclusion of mental health and substance use disorders as covariates, as most patients with PDs sustain high levels of comorbidity, thus reducing the possibility of confounding.

The study did not include all possible cost items in the cost interview, such as costs to society due to criminality and home care costs. Especially costs of crime to society could be significant among some patients with PDs, in particular antisocial PD, [75]. In a recent study of the economic cost of crime in North America attributable to people with psychopathic personality disorder (PPD), which can be conceptualized as a more severe version of antisocial personality disorder [76], the estimated PPD-related costs of crime ranged from $245.50 billion to $1,591.57 billion in the United States and $12.14 billion to $53.00 billion in Canada. These results suggest that PPD may be associated with a substantial economic burden as a result of crime in North America [76]. In our study, 2.4% of the patients with PDs were diagnosed with antisocial PD, and their contribution to societal cost may have been underestimated due to the omission of criminality costs. Furthermore, we did not differentiate between secure forensic settings and ordinary psychiatric hospitals when patients were interviewed about health service use, due to the fact that official cost statistics do not differentiate between these types of hospital admission. As a secure forensic setting typically has a higher personnel rate and provide enhanced services, the costs would regularly be somewhat higher than ordinary psychiatric hospital costs, leading to a possible underestimation of the impact on costs of patients with antisocial PD.

Diagnostic reliability was not investigated directly in this study, and inadequate consistency could lead to biased estimators and reduced statistical significance. However, the interviewers had received systematic training in diagnostic interviews and principles, and all diagnoses were set or evaluated by a specialist in psychiatry or clinical psychology. Furthermore, in a former study using the Structured Clinical Interview for DSM-IV (SCID-II—the previous version of SCID-5-PD) within the same Network, reliability was investigated and acceptable diagnostic reliability was indicated [26].

The use of health services data and workforce participation are collected retrospectively, and may be susceptive to recollection bias. The relatively short measurement period of 6 months was set to reduce this, but the limited range may have reduced the variation in the sample at the same time. This may again have led to reduced levels of explained variance and stability, especially in the productivity loss model, with many responses at both ends of the scale.

In the present study, we have not included an investigation of cost implications of dimensional measures of PD severity or different aspects of personality functioning, which could possibly differentiate the findings. This should be a topic for further research.

## Conclusion

The main result of this study was that no specific PD had a unique contribution to the generally high level of societal costs, in a model including all DSM-5 PDs and comorbid mental health and substance use disorders. The low level of explained variance in this model, albeit comparable to other studies, implies that distinct PD-categories may not be the best predictors of societal costs, and dimensional models of PD should be investigated in future research. Although BPD was associated with increased levels of health service costs, and BPD, avoidant PD, and unspecified PD were associated with enhanced productivity loss, the differential effects of these specific PDs were small, as reflected in the small effect sizes in all regression models. In order to reduce societal costs, the importance of developing and implementing effective treatments for a broad range of PDs, not only BPD, is implied by the results of this study.

## Data Availability

Due to restrictions imposed by the Regional Medical Ethics Committee regarding patient confidentiality, data are available upon request. Requests for data may be sent to the hospital's Privacy and Data Protection Officer at: personvern@ous-hf.no.

## Footnotes

1 Note: Model I is log transformed, while model II and III are not, thus model III (the sum of health service costs and productivity loss) can be non-significant even if both models of the components are significant. Some nuances may also be lost at the aggregate level, compounding relatively uncorrelated variables.
